# A systematic review of the psychometric properties of Quality of Life measures for school aged children with cerebral palsy

**DOI:** 10.1186/1471-2431-10-81

**Published:** 2010-11-09

**Authors:** Stacey Carlon, Nora Shields, Katherine Yong, Rose Gilmore, Leanne Sakzewski, Roslyn Boyd

**Affiliations:** 1 School of Physiotherapy, La Trobe University, Bundoora, Victoria, Australia; 2 Florey Neurosciences Institute, Austin Repatriation Hospital, Heidelberg West, Victoria, Australia; 3 Queensland Cerebral Palsy and Rehabilitation Research Centre, School of Medicine, The University of Queensland, Herston, Queensland, Australia; 4 Musculoskeletal Research Centre, La Trobe University, Bundoora, Victoria Australia; 5 Department of Paediatrics and Child Health, The University of Queensland, Royal Children's Hospital, Herston, Queensland, Australia

## Abstract

**Background:**

This systematic review aimed to evaluate the psychometric properties and clinical utility of all condition specific outcome measures used to assess quality of life (QOL) in school aged children with cerebral palsy (CP).

**Methods:**

Relevant outcome measures were identified by searching 8 electronic databases, supplemented by citation tracking. Two independent reviewers completed data extraction and analysis of the measures using a modified version of the CanChild Outcome Measures Rating Form.

**Results:**

From the 776 papers identified 5 outcome measures met the inclusion criteria: the Care and Comfort Hypertonicity Questionnaire (C&CHQ), the Caregiver Priorities and Child Health Index of Life with Disabilities (CPCHILD), CP QOL-Child, DISABKIDS and PedsQL 3.0 CP Module. There was evidence of construct validity for all five measures. Content validity was reported for all measures except PedsQL 3.0. The CPCHILD and CP QOL-Child were the only outcome measures to have reported data on concurrent validity. All measures, with the exception of one (C&CHQ) provided evidence of internal reliability. The CPCHILD and the CP-QOL-Child had evidence of test-retest reliability and DISABKIDS had evidence of inter-rater reliability. There were no published data on the responsiveness of these outcome measures.

**Conclusions:**

The CPCHILD and the CP QOL-Child demonstrated the strongest psychometric properties and clinical utility. Further work is needed, for all measures, on data for sensitivity to change.

## Background

Cerebral Palsy (CP) defines a group of conditions, arising from an injury to the developing brain and occurs in 2.0 children per 1000 live births [1]. In addition to the disturbances of movement and posture including spasticity, muscle weakness and reduced coordination, common impairments of children with CP include disturbances of sensation, perception, cognition, communication, behaviour, epilepsy, and secondary musculoskeletal problems [2]. Reduced activity levels and participation restrictions due to these impairments may lead to a reduced quality of life (QOL), compared to their typically developing peers [3-5]. The World Health Organization (WHO) defines QOL as "an individual's perception of their position in life in the context of the culture and value systems in which they live, and in relation to their goals, expectations, standards and concerns"[6]. Quality of life is also defined as a person's feelings of well-being across many domains including physical, social, emotional and spiritual aspects of life [7]. Outcome measures that purport to evaluate QOL should take a broad approach to measuring well-being, and not just measure on the functional domain [8,9].

Quality of life measures can either be generic (that is, measure well-being of any child, typically developing or with a disability), for example the KIDSCREEN [10], or be condition specific (that is, focuses specifically on a defined population and tailors its questions to the issues that might impact on the QOL of that population) [7]. Condition specific measures of QOL have a role to play for children with cerebral palsy as they include all domains unique to the population group, such as physical functioning, adaptive equipment as well as psychosocial domains [7]. When available, a condition specific measure is preferable to a generic measure in that it is able to address the aspects of life which are unique to a given population group. For example, a CP specific outcome measure would be able to explore a child's feelings surrounding any adaptive equipment that they require, or feelings surrounding medical, surgical or therapeutic interventions, whereas these topics would be beyond the scope of a generic outcome measure, thus omitting potentially important aspects of daily life from review. This review will focus on the role of condition specific QOL measures as it is thought that such instruments offer a greater depth of insight into the QOL of children with CP [11]. As with all outcome measures, measures of QOL should be valid, reliable, and responsive to change for the population of interest while also being easy to complete, analyse and access [12].

Increasingly, research and clinical practice is focusing on interventions not only to improve biomechanical alignment or functional outcomes for children with CP but also to positively improve QOL. Clinicians need to utilise outcome measures that accurately assess QOL in these children to provide evidence that their management strategies are influencing a child's QOL. The aim of this review therefore was to systematically identify all available condition specific QOL outcome measures for school aged children with CP, and to evaluate their psychometric properties and clinical utility.

## Methods

### Search Strategy

Articles were identified from a systematic search of the following computerized bibliographic databases: CINAHL (1982-October 2009), Medline (1950-October 2009), EMBASE (1988-October 2009), AMED (1985-October 2009), PsychINFO (1967-October 2009), PEDro (1929-October 2009), the Cochrane Library and ERIC (1966-October 2009). The keywords used were "quality of life," "cerebral palsy," "children" and "outcome measure," along with relevant MeSH terms and synonyms and searches were combined to obtain the final yield. Further articles were identified through citation tracking using the Web of Science databases, manual checking of the reference lists of included articles, and consultation with experts in the field.

### Inclusion/Exclusion Criteria

Outcome measures were included if they were designed to measure quality of life, according to the WHO definition, in children with CP aged 4 to 18 years and were written in English. This lower age limit was chosen so as to make certain a correct diagnosis of CP.

Outcome measures were excluded if they were a generic QOL outcome measure or measured a construct other than QOL such as functional or health status and therefore did not meet the WHO definition. Functional status was described as a child's capacity to fulfill the requirements of day-to-day living, including societal contributions and personal up-keep [8], for example the Pediatric Outcomes Data Collection Instrument. Health status included a child's level of health versus ill health, incorporating symptoms and dysfunctions and disorder management [8], for example the Child Health and Illness Profile. It is important to include only measures based on a theoretical construct of QOL as measures of function or health status; while related, do not specifically address the required definitional criteria of QOL.

Two independent reviewers (SC, KY) excluded papers from the initial search yield on the basis of title and abstract. Full text articles were sourced where the title and abstract did not provide enough information about whether the inclusion criteria were met. Any disagreements over which papers to include or exclude were discussed with a third reviewer (RG) until a consensus was reached.

### Quality Assessment

Quality assessment was completed by the two independent reviewers (SC, RG), using a modification of the CanChild Outcome Measures Rating Form and Guidelines [13,14], an assessment scale that evaluates the psychometric properties and clinical utility of outcome measures (Appendix A). The modifications for the current review, were (1) the sections of the rating form focusing on the ICF domains of an individual's ability to perform activities and to participate in society were omitted and (2) the remaining items were retabulated for ease of use by reviewers. The performance of the omitted items does not relate as directly to QOL as do the feelings and perceptions regarding these life areas. The modified form also considered the quality of the publications from which information on each outcome measure was sourced.

The modified rating form consisted of eleven items which were applied to assess clinical utility (ease of interpretation, feasibility, and how acceptable the measure was to assessors and respondents); and psychometric properties; including scale construction, standardization, reliability (internal consistency, retest, and inter-rater), validity (content, concurrent and construct) and responsiveness.

Evidence for each aspect of quality measurement was scored as excellent (3 points), adequate (2 points), poor (1 point), or a no evidence available option was given (zero points). To be allocated 3 points there must have been at least two high quality published papers reporting strong evidence for the outcome measure in question. A score of 2 points required 1-2 well-designed studies with adequate to excellent results. If poor quality studies were conducted; or poor levels of evidence shown, a score of 1 was given. Such cut-offs were put into place so that only outcome measures which had a body of high-quality evidence of psychometric properties, along with high levels of clinical utility would be considered as excellent.

### Data Extraction and Analysis

Data were extracted by two reviewers (SC, RG) using a standardized form on the theoretic construct of the outcome measure, the respondent(s), standardization measures available (for example a manual or administration instructions), the target population, and the cost and training involved. Information on validity, reliability and responsiveness was also tabulated for each of the outcome measures by the two reviewers (SC, RG), based on the evidence sourced from publications. The quality of psychometric properties and clinical utility were then ranked by the two reviewers.

## Results

**Figure 1:**
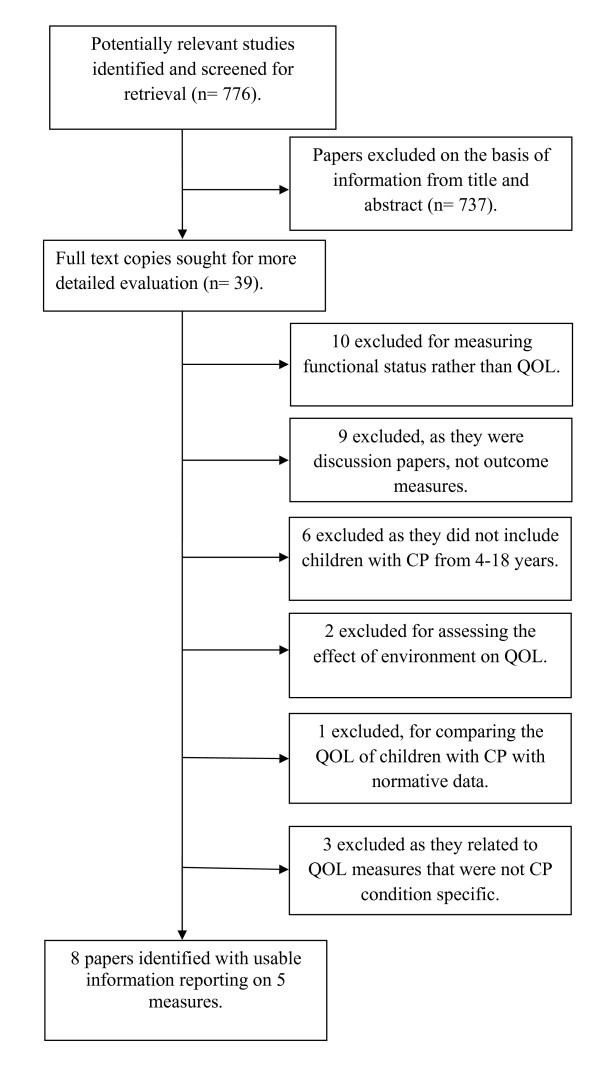
**Processes performed for outcome measure and paper selection**.

The systematic search yielded 776 references of which 39 papers remained after review of the title and abstract. The full text of these papers were retrieved and assessed by two independent reviewers (SC, KY) and a further 31 papers were excluded (see Figure 1). Eight papers reporting the five outcome measures met the full inclusion criteria (Table 1). The condition specific QOL outcome measures for school aged children with CP identified were the Care and Comfort Hypertonicity Questionnaire (C&CHQ) [15], the Caregiver Priorities and Child Health Index of Life with Disabilities (CPCHILD) [16,17], CP QOL-Child [18], DISABKIDS [19-21], and the PedsQL 3.0 Cerebral Palsy Module [22] (Table 1).

**Table 1:** Data Extraction Summary Table

Study	Outcome Measure	Sample Size Total(% female)	Mean age Years, months; SD (range)	Motor type and Distribution	Functional Severity GMFCS Levels (I-V)
**McCoy, et al. 2006**	C&CHQ	47 (38)	10 ± 4 (3.1-21.1)	Spasticity/dystonia	-
**Narayanan, et al. 2006**	CPCHILD	77 (42)	13.5 ± 3.4 (5-18)	-	I: 16% II: 4% III: 13% IV: 14% V: 53%
**Narayanan, et al. 2007**	CPCHILD	67 (37)	Ambulatory CP: 8.3(5-18) Non-Amb CP: 10.2 (5-18)	-	I: 18% II:4% III: 21% IV: 16% V: 40%
**Waters, et al. 2007**	CP QOL-Child	205 (45)	8.5 (4-12)	-	I: 18% II: 28% III: 14% IV: 11% V: 27%
**Baars, et al. 2005**	DISABKIDS (condition specific module)	21	(overall pop.) 12.5 ± 2.5 (8-16)	-	-
**Petersen, et al. 2005**	DISABKIDS (chronic generic module)	21*	(overall pop.) 12.5 ± 2.5 (8-16	-	-
**Schmidt, et al. 2006**	DISABKIDS (chronic generic module)	91#	12.2 ± 2.8 (8-16)	-	Mild: 24.4% Moderate: 48.8% Severe: 26.8%
**Varni, et al. 2006**	PedsQL 3.0	241 (44)	8.1 ± 4.4 (2-18)	Hemiplegia = 55 Diplegia = 84 Quadriplegia = 85 Ataxia = 7 Diagnosis unavailable = 14	I: 11% II: 16% III: 35% IV: 18% V: 15%

*Note*. Hyphens (-) indicate no published research, or information not available. *CP population as a part of a study with 360 children with chronic conditions (asthma, arthritis, cerebral palsy, cystic fibrosis, dermatitis, diabetes, and epilepsy), 52% female. # CP population as a part of a study with 1153 children with chronic conditions (asthma, arthritis, cerebral palsy, cystic fibrosis, dermatitis, diabetes, and epilepsy), 48% female.

Three of the five measures used a grounded theory and had their development based on the topics and themes garnered from interviews with parents of children with cerebral palsy, as well as health and care professionals (CPCHILD, CP QOL-Child, DISABKIDS). Importantly, the process for developing both the CP QOL-Child and the DISABKIDS included discussion and consultation with children who have CP. The basic premise for the C&CHQ was sourced from an earlier outcome measure, The Caregiver Questionnaire [23]. Information surrounding the development of the PedsQL 3.0 states that research and clinical experience were the primary basis of the questionnaire formulation. Additional information was gathered through focus groups and interviewing, though details of the group participants were not provided.

All outcome measures were designed to be completed by parents (parent report), and three of the five measures also had a child report version, administered to children eight years and older; CP QOL-Child [18], DISABKIDS [19] and PedsQL 3.0 [22].

In the eight studies reporting the measures only four papers reported on the GMFCS levels of participants [16-18,22]. Of those reported, there was a greater representation of children with skills classifications of GMFCS level V (28%), and GMFCS level III (24%). Children with a skills classification of GMFCS levels I and II accounted for 34% of the reported population which is lower than the 50% distribution reported in the CP Register Australia's 2009 inaugural report, detailing the 1993-2003 birth cohort [24].

### Quality Assessment

Quality assessment found two outcome measures (CPCHILD, CP QOL-Child) that were considered to be of a high standard based on the aforementioned ratings. Two measures (DISABKIDS and PedsQL 3.0) were considered to be moderately constructed, while the C&CHQ was scored as a poor outcome measure on the quality scale used (Table 2).

**Table 2:** Quality Assessment Summary

Outcome Measure	Clinical Utility (3)	Scale Construction (3)	Standardisation (3)	Reliability (6)	Validity (15)	Overall Utility (3)
**C&CHQ**	1	1	1	0	4	1
**CPCHILD**	3	2	3	4	9	3
**CP QOL-Child**	3	3	3	4	9	3
**DISABKIDS**	3	2	3	4	7	2
**PedsQL 3.0**	3	1	2	3	6	2

*Note*. Total possible score for each domain in brackets.

Key: C&CHQ = Care and comfort hypertonicity questionnaire. CPCHILD = Caregiver priorities and child health index of life with disabilities. Clinical utility relates to clarity of instructions. Scale construction includes assessment of relevant characteristics of construct and theoretical construct questionnaire developed under. Reliability includes intra-rater, retest and internal consistency. Validity includes content, construct, criterion and responsiveness. Overall utility combines all relevant information available for outcome measures.

### Theoretical constructs of outcome measures

The definition of QOL as described by Bjornson and McLaughlin [7] compromised two components; QOL should be assessed over broad domains, and also be a measure of well-being. The CP QOL-Child was the only measure to base its development on this theoretical construct and measured well-being. Other identified outcome measures phrased questions in the negative, thus, not measuring well-being, but rather, ill-being. All five identified measures fulfilled the requirement to measure QOL over a number of areas of life (Table 2).

### Evidence of Validity, Reliability and Responsiveness

The available data on the psychometric properties of the five included measures are presented in Table 3. There was evidence of construct validity for all five measures. Three studies used the Gross Motor Function Classification system (GMFCS) to assess construct validity [16,17,22]. The PedsQL 3.0 reported discriminant validity on the basis that typically developing children scored higher on average than children with CP. Four of the five outcome measures reported content (face) validity (C&CHQ, CPCHILD, CP QOL-Child and DISABKIDS). Both the CPCHILD and CP QOL-Child reported data on concurrent validity.

**Table 3:** Characteristics of selected quality of life measures

Outcome Measure	Age	Domains (no of items)	Content Validity	Concurrent Validity	Construct (convergent/divergent) Validity	Internal Reliability	Retest (ICC) Reliability
**Care & comfort hypertonicity questionnaire (C&CHQ)**	-	1) Personal care (8) 2) Positioning/Transfer (7) 3) Comfort (5) 4)Interaction/Communication (7)	Retrospective analysis of patient notes and interviews of experts.	-	Higher scores were referred to ITB therapy, lower scores to Botulinum toxin.	-	-
**Caregiver priorities and child health index of life with disabilities (CPCHILD)**	5-12y	1) Personal care (8) 2) Positioning, transferring & mobility(8) 3) Comfort emotions and behaviour (9) 4) Communication and social interaction (7) 5) Health (3) 6) Overall quality of life (1)	Caregiver rated importance of items × = 3.95 on 6 point ordinal scale (SD 0.63, range 2.67-4.90)	Domains moderately correlated to those of CHQ and PEDI	Higher GMFCS scores correlated with higher CPCHILD scores (worse outcome). Convergent (Spearman's ρ): (w. PEDI) Self Care 0.607 Mobility 0.619 Social Function 0.518	α = 0.74-0.93 across all domains	0.97 total questionnaire. 0.88-0.96 across the 6 domains. (95% CI 0.88-0.99). 0.85 (95% CI: 0.68-0.93)
**CP QOL-Child**	4-12y	1) Social well-being and acceptance 2)Participation and physical health 3)Functioning 4)Emotional well-being 5)Pain and impact of disability 6)Access to services 7)Family Self-report: 53 items. Parent Proxy: 66 items	Domains determined in consultation with children with CP and their parents (28 families)	Domains moderately correlated to those of KIDSCREEN and CHQ	Global QOL r = 0.18-0.62 Global Health r = 0.21-0.56	α = 0.74-0.92 (caregivers) (n = 205) α = 0.80-0.90 (child-self report) (n = 53)	0.76-0.89
							
DISABKIDS*	4-16y	1) Impact of disease (10) 2) Communication (2)	Domains determined in consultation with children and their parents (9 families).	-	Discriminate: was more able to differentiate between levels of disability than CHQ and KINDL	α = 0.71-0.91 Inter-rater (concordance) 0.14-0.84 (across scales)	-
**PedsQL 3.0 CP Module**	2-18y	1) Daily activities(9) 2) School activities (4) 3) Movement and balance (5) 4) Pain and hurt (4) 5) Fatigue (4) 6) Eating activities (5) 7) Speech and communication (4)	-	-	Discriminate: Able to distinguish between typically developing children and children with CP.	Child Self-report: 0.77-0.93	-

*Note*. *DISABKIDS questionnaire for young people with cerebral palsy. Hyphens (-) indicate no published research, or information not obtained. PPC: Pearson's Correlation Coefficient. CHQ: Child health questionnaire. KIDSCREEN: a generic quality of life measure. KINDL: a generic health-related quality of life measure. PEDI: Pediatric Evaluation of Disability Inventory.

Four of the five outcome measures (CPCHILD, CP QOL-Child, DISABKIDS, and PedsQL 3.0) reported data on the internal reliability of the domains (Table 3). There was evidence for retest reliability on two measures (CPCHILD, CP QOL-Child), and evidence of inter-rater reliability for one measure (DISABKIDS).

No information was available on measurement error or responsiveness of any of the included measures to change. McCoy et al. [15] reported on the sensitivity of the C&CHQ, noting an improvement of QOL score in children when their dose of intrathecal baclofen was increased to reduce spasticity. Varni et al. [22] reported on sensitivity of the PedsQL 3.0 measure, finding that children with a distribution of quadriplegia had a lower HRQOL than children with hemiplegia and diplegia. It was also reported that children with lower GMFCS scores representing a higher functioning ability (GMFCS I and II) demonstrated an increased HRQOL. Only one outcome measure (DISABKIDS) reported data on floor (0.3% in 'overall' domain) and ceiling (2.3% in 'overall' domain and 1.7% in 'physical' domain) effects in the group sampled.

### Clinical Utility

Analysis of clinical utility in the five included measures found that four reported information on completion time (CPCHILD, CP QOL-Child, DISABKIDS, PedsQL 3.0) (Table 4). The CPCHILD was expected to take 20-30 minutes to complete, the CP QOL-Child 15-25 minutes, the DISABKIDS 20 minutes and the PedsQL 3.0 five minutes to complete. The cost of administering the outcome measures was available for all of the five instruments (Table 4). The C&CHQ, CPCHILD and the CP QOL-Child were available free of charge for all users.

**Table 4:** Clinical utility of the selected QOL measures for children with CP

Outcome Measure	Questionnaire completed by	Completion Time (minutes)	Cost	Outcome Measure Description
**C&CHQ**	Caregiver/Parent	-	No cost for use of questionnaire. No manual available.	Designed to capture QOL improvements as a result of reduced tone post intrathecal baclofen (ITB) treatment
**CPCHILD**	Caregiver/Parent (5-12 yrs)	20-30	No cost for questionnaire or manual. Registration requested.	A measure of health status and well- being for children with severe cerebral palsy, developed to measure effectiveness of interventions
**CP QOL-Child**	Caregiver/Parent (4-12yrs) Child (9-12 yrs)	15-25	No cost for questionnaire or manual. Registration requested.	A condition specific outcome measure intended to gauge and assess QOL changes in children with cerebral palsy
**DISABKIDS**	Caregiver/Parent (4-16 yrs) Child (4-16 yrs)	-	Non-funded & Government funded academic research*: free Large non-commercial organisation research and evaluation*: free Commercial studies*: 1000€ - 5000€	Intended to measure HRQOL and assess burden of disease in children and adolescents
**PedsQL 3.0**	Caregiver/Parent (2-18 yrs) Child/Adolescent (5-18 yrs)	5	Non-funded academic: free Funded academic: $600US per study Large Non-Commercial: $1600US Large commercial $5600-$20,600US	Developed to assess HRQOL in a population of children and adolescents with cerebral palsy

*Note*. Hyphens (-) indicate no published research, or information not available. *Purchase of 50€ manual and questionnaires required. #PedsQL standardised guidelines for administration available at no cost.

## Discussion

Ideally, therapists and researchers hoping to assess QOL in children with CP should have a choice of valid, reliable, easy to administer, low cost instruments, suited to the cultural and societal background of the children involved. This review reported and evaluated the psychometric properties and clinical utility of the condition specific outcome measures currently used to measure QOL in children with CP. Several areas were identified where further research is required including, data supporting concurrent validity, retest reliability and the responsiveness of the outcome measures. Based on the collected psychometric and clinical utility data of the included studies, the CPCHILD and the CP QOL-Child were the strongest outcome measures for evaluating QOL in school aged children with CP. The CP QOL-Child was the only outcome measure to have been developed with a firm theoretical underpinning of QOL.

In the process of identifying and evaluating available outcome measures, the agreement of each to the accepted definition of QOL was assessed. Only one outcome measure fulfilled the two-part definitional criteria of QOL being measured across broad domains and being a measure of well-being (CP QOL-Child). It is recommended that clinicians and researchers utilise outcome measures that fulfil the requirements of the accepted definition. Self-evaluation of well-being has been theorised to stem from positive internal attitudes and feelings [25]; accordingly, outcome measures aiming to assess QOL should be phrased to measure well-being (for example CP QOL-Child), rather than shaping questions assuming ill-being (for example, DISABKIDS). Furthermore, the assumption of difficulty, challenge and ill-being in QOL outcome measures for children with CP is not in keeping with the principles and the purposes of these instruments. Quality of Life instruments purpose to measure and explore feelings and daily life experiences, and should not formulate questions which assume to know a person's state of being, simply due to a condition they have.

All five outcome measures available to measure QOL in school aged children with CP have limited evidence of validity. In three studies [16,17,22] the GMFCS was used to provide evidence for construct validity. Construct validity refers to the degree to which an outcome measure correlates to other measures, in a style uniform with theoretically gained principles concerning the measured concept [26]. When a QOL measure has a large focus on functionally based questions, it can be expected that as GMFCS scores increase (representing a greater gross motor impairment), the level of reported QOL is reduced, as functional tasks become more difficult [27]. This correlation may not be seen between a generic QOL measure and the GMFCS, as domains may not cover relevant aspects of life for a child with cerebral palsy. The GMFCS is a measure of motor function ability and not a QOL measure, thus correlations between QOL outcome measures and the GMFCS do not necessarily result in a valid QOL measure, but rather a measure that may differentiate across the spectrum of functional severity. Similarly, discriminant validity for the PedsQL 3.0 was reported on the basis that typically developing children scored higher on average than children with CP [22]. The PedsQL 3.0, as a QOL outcome measure has an overemphasis on the functional domains, for example, of the 35 items in the 13-18 year old parent report, 23 items (66%) relate to functional tasks. Participants are asked about the difficulty of completing tasks in their daily life, and considering that poor physical function does not necessarily correlate with an overall poor QOL [28,29] this method has limitations.

With the exception of the CPCHILD and the CP QOL-Child, outcome measures evaluated in this review did not provide evidence to support their concurrent validity. While there is no agreed 'gold standard' QOL measure to compare these CP condition specific outcome measures to, researchers could analyse the correlations between their outcome measure and a combination of other validated and reliable QOL measures. Accordingly, the CP QOL-Child was validated against the KIDSCREEN, a generic measure of QOL [18]. Such research needs to be conducted to further strengthen the case for condition specific QOL measures.

As QOL is a personal perspective of an individual's well-being, testing reliability may be seen by some as problematic. In theory, it should be possible; with the absence of any major life changes, the results of consecutive applications of a QOL questionnaire should be consistent. There was no evidence for re-test reliability for three of the five QOL outcome measures; the C&CHQ, DISABKIDS and PedsQL 3.0. An assessment of re-test reliability for both the child and parent report was conducted for the CP QOL-Child, with applications of the questionnaire occurring at baseline and two weeks. Additional questions were administered after the two week period to gauge for any major life events during that time. Given this limited evidence for re-test reliability, it would be hard for clinicians and researchers using these outcome measures to determine if changes have really occurred following an intervention.

This review identified that there is a lack of data on responsiveness, measurement error and minimally clinically significant difference scores for all of the measures. While the C&CHQ presented data on sensitivity to change, there was a potential bias in that the parents reporting the scores knew of the change in medical management. As rehabilitation studies are rarely masked to treatment allocation, it is problematic for parents to report perceived QOL without knowledge of treatment allocation, introducing bias. Information on sensitivity and responsiveness is important as increasingly QOL measures are being used in research studies. Until there is empirical evidence on the sensitivity to change and measurement error of these outcome measures, we cannot properly interpret results obtained from these outcome measures. The condition specific outcome measures for measuring QOL have only recently been developed, thus information regarding the appropriate minimally clinically significant change for each of the measures is now needed. It needs to be clear whether a change in domain or total score correlates to a clinically important change in QOL.

Ceiling and floor effect sizes were reported for only one outcome measure (DISABKIDS chronic generic). This scale was designed as a 'chronic generic measure', including not only children with CP, but other paediatric conditions, thus, some questions may not be as appropriate as in other outcome measures. It is a positive indication of the validity of the given outcome measures if such effects did not generally occur. It would be expected that ceiling and floor effects would only occur if an outcome measure was inappropriately used on a population, for example a commonly erroneously used health status measure such as the PODCI, which is often referred to as a measure of QOL [30].

During compilation of the studies in this review, the lack of reported demographic details of the population of interest in published works was evident. One short-coming was the lack of information regarding the cognitive abilities of children taking part in the self reporting of QOL. Children with CP may have cognitive impairments and such information is vital to researchers when deciding on whether an outcome measure to assess QOL is appropriate for a particular client or not. The CPCHILD specifically focused on children with greater physical impairment (children with GMFCS classifications III to V) and relied on parents' report of their child's QOL [16].

Another point of interest was the wide age ranges that were used in four of the studies [15-17,22]. When a small population is dispersed over such a broad age range and a spectrum of severity (GMFCS levels), questions of validity of the child report of results may arise. Consideration of the appropriateness and relevance of questions across the spectrum of age and severity is needed as there may be incredible variation in life experiences present between children of different ages and GMFCS levels. It is questionable whether items relevant to a group of primary school aged children would be equally relevant and have the same priority as QOL questions for adolescent aged children. It is recommended that condition specific tools of QOL are developed and validated for specific age bands (child, teenager and adult), to capture the most meaningful and important data.

A limitation of this review was that potentially relevant articles were excluded if they were not written in the English language. Other limitations were the relatively recent development of the QOL outcome measures, resulting in a paucity of data on the ability of the measures to detect clinically important changes in QOL due to interventions.

## Conclusions

Taking into account the evidence for reliability and validity, the ease of access, the relatively quick completion time, and the free availability, the CP QOL-Child and the CPCHILD were found to be the strongest measures of QOL in children with CP. The CP QOL-Child was the only measure which wholly fulfilled the definitional criteria of QOL. As yet however, there is no data published on sensitivity and limited data on the child report questionnaire for the CP QOL-Child. More broadly, the term "Quality of Life" is used with much inconsistency. Both research and clinical practice would benefit from the uniform understanding and use of an agreed, consensus driven definition.

## Competing interests

Contributing author RB was involved in the consultation processes which lead to the development and psychometric testing of the CP QOL-Child questionnaire. This bias was eliminated by RB not being involved with the quality analysis of the questionnaires.

## Authors' contributions

SC assisted in developing the search strategy and modifying the quality analysis tool, conducted the search, screened publications for inclusion, applied inclusion and exclusion criteria, performed data extraction and quality analysis and drafted the manuscript. NS contributed to developing the study methodology, assisted in developing the search strategy and modifying the quality analysis tool, oversaw the assessment of each measure and provided input at each stage of the drafting of the review. KY screened publications for inclusion and applied the inclusion and exclusion criteria. RG assisted with application of inclusion and exclusion criteria, performed data extraction and quality analysis. LS provided methodological input, background information and clinically applicable support. RB provided background information, assisted in developing the review methodology, assisted in developing the search strategy and modifying the quality analysis tool and provided input at each stage of the drafting of the review. All authors have been involved in the revision of this document and have approved the final manuscript.

## Appendix A

**Table:** Quality Analysis (Adapted) Outcome Measures Rating Form CanChild Centre for Disability Research, Institute of Applied Health Sciences, McMaster University.

CLINICAL UTILITY	Clarity of Instructions	□ Excellent (clear, comprehensive, concise, and available) □ Adequate (clear, concise, but lacks some information) □ Poor (not clear and concise or not available)
**SCALE CONSTRUCTION**	Item Selection	□ Excellent (included all relevant characteristics of attribute based on comprehensive review and survey of experts) □ Adequate (included most relevant characteristics of attribute) □ Poor (convenient sample of characteristics of attribute, or questionnaire not available)
**STANDARDIZATION**	Manual	□ Excellent: published manual which outlines specific procedures for administration; scoring and interpretation; evidence of reliability and validity □ Adequate: manual available and generally complete but some information is lacking or unclear regarding administration; scoring and interpretation; evidence of reliability and validity □ Poor: no manual available or manual with unclear administration; scoring and interpretation; no evidence of reliability and validity

**RELIABILITY**	Rigor of standardization studies for reliability	□ Excellent: > 2 well-designed reliability studies completed with adequate to excellent reliability □ Adequate: 1 to 2 well-designed reliability studies completed with adequate to excellent reliability □ Poor: reliability studies poorly completed, or reliability studies showing poor levels of reliability □ No evidence available

	Reliability Information	Type of Reliability Statistic Used Value Rating (excellent, adequate or poor) NB Excellent: > .80 Adequate:.60 -.79, Poor: < .60

**VALIDITY**	Rigor of standardization studies for validity	□ Excellent: more than 2 well-designed validity studies supporting the measure's validity □ Adequate: 1 to 2 well-designed validity studies supporting the measure's validity □ Poor: validity studies poorly completed or did not support the measure's validity □ No evidence available

	Content Validity	□ Excellent: judgmental or statistical method (e.g. factor analysis) was used the measure is comprehensive and includes items suited to the measurement purpose *Method*: □ judgmental □ statistical □ Adequate: has content validity but no specific method was used □ Poor: instrument is not comprehensive □ No evidence available

	Construct Validity	□ Excellent: more than 2 well designed studies have shown that the instrument conforms to prior theoretical relationships among characteristics or individuals □ Adequate: 1 to 2 studies demonstrate confirmation of theoretical formulations □ Poor: construct validation poorly completed, or did not support measure's construct validity □ No evidence available

	Criterion Validity	□ Concurrent □ Predictive Criterion Measure used: □ Excellent: > 2 well-designed studies showing adequate agreement with a criterion or gold standard □ Adequate: 1-2 studies demonstrate adequate agreement with a criterion or gold standard measure □ Poor: criterion validation poorly completed or did not support measure's criterion validity □ No evidence available

**RESPONSIVENESS**		□ Excellent: more than 2 well-designed studies showing strong hypothesized relationships between changes on the measure and other measures of change on the same attribute. □ Adequate: 1-2 studies of responsiveness □ Poor: studies of responsiveness poorly completed or did not support the measure's responsiveness □ No evidence available

**OVERALL UTILITY**		□ Excellent: adequate to excellent clinical utility, easily available, excellent reliability and validity □ Adequate: adequate to excellent clinical utility, easily available, adequate to excellent reliability and adequate to excellent validity □ Poor: poor clinical utility, not easily available, poor reliability and validity

## Pre-publication history

The pre-publication history for this paper can be accessed here:

http://www.biomedcentral.com/1471-2431/10/81/prepub
